# Cerium Oxide Nanoparticles Re-establish Cell Integrity Checkpoints and Apoptosis Competence in Irradiated HaCat Cells via Novel Redox-Independent Activity

**DOI:** 10.3389/fphar.2018.01183

**Published:** 2018-10-16

**Authors:** Fanny Caputo, Anna Giovanetti, Francesca Corsi, Vittoria Maresca, Stefania Briganti, Silvia Licoccia, Enrico Traversa, Lina Ghibelli

**Affiliations:** ^1^Department of Chemical Science and Technologies, University of Rome Tor Vergata, Rome, Italy; ^2^Department of Biology, University of Rome Tor Vergata, Rome, Italy; ^3^ENEA SSPT-TECS-BIORISC, Rome, Italy; ^4^San Gallicano Dermatological Institute IRCCS, Rome, Italy; ^5^School of Materials and Energy, University of Electronic Science and Technology of China, Chengdu, China

**Keywords:** cerium oxide nanoparticles, apoptosis, radio-sensitization, DNA damage response, anticancer therapy, DNA integrity checkpoints

## Abstract

Cerium oxide nanoparticles (CNPs) are potent radical scavengers protecting cells from oxidative insults, including ionizing radiation. Here we show that CNPs prevent X-ray-induced oxidative imbalance reducing DNA breaks on HaCat keratinocytes, nearly abating mutagenesis. At the same time, and in spite of the reduced damage, CNPs strengthen radiation-induced cell cycle arrest and apoptosis outcome, dropping colony formation; notably, CNPs do not possess any intrinsic toxicity toward non-irradiated HaCat, indicating that they act on damaged cells. Thus CNPs, while exerting their antioxidant action, also reinforce the stringency of damage-induced cell integrity checkpoints, promoting elimination of the “tolerant” cells, being in fact radio-sensitizers. These two contrasting pathways are mediated by different activities of CNPs: indeed Sm-doped CNPs, which lack the Ce^3+^/Ce^4+^ redox switch and the correlated antioxidant action, fail to decrease radiation-induced superoxide formation, as expected, but surprisingly maintain the radio-sensitizing ability and the dramatic decrease of mutagenesis. The latter is thus attributable to elimination of damaged cells rather than decreased oxidative damage. This highlights a novel redox-independent activity of CNPs, allowing selectively eliminating heavily damaged cells through non-toxic mechanisms, rather reactivating endogenous anticancer pathways in transformed cells.

## Introduction

Cerium oxide nanoparticles (CNPs) are attracting much interest in biomedical applications for their anti-oxidant properties provided by the Ce^3+^/Ce^4+^ redox couple on the nanoparticles surface, which combine catalase- and superoxide dismutase (SOD)-mimetic activities scavenging hydrogen peroxide and superoxides in a self-regenerating manner (Das et al., 2013). Coupled with high biocompatibility (Park et al., 2008; O’Brien and Cummins, 2010), protection from oxidative damage and cell death (Celardo et al., 2011b), and amelioration of oxidative pathologies (D’Angelo et al., 2009), the peculiar antioxidant properties of CNPs are considered very promising pharmacological tools (Walkey et al., 2015). In particular, CNPs have been shown to act as radio-protective agents (Tarnuzzer et al., 2005), an ability that is generally attributed to scavenging of radiation-induced oxidative stress (Colon et al., 2010).

Cerium oxide nanoparticles show intriguing potential anticancer effects (Corsi et al., 2018), reducing tumor growth in animal models (Giri et al., 2013), and ameliorating malignant features of cancer microenvironment such as neo-angiogenesis (Giri et al., 2013; Lord et al., 2013) and stroma-tumor altered communications (Alili et al., 2011). CNPs show additional intriguing anticancer properties: they ameliorate the effect of radiotherapy increasing killing of tumor cells (Wason et al., 2013), a major issue considering that cancer cell radio-resistance is a main obstacle to successful radiotherapy; moreover, CNPs seem to be preferentially toxic in cancer vs. non-transformed cells (Sack et al., 2014). The mechanisms of such effects are still unclear: indeed, they cannot be satisfactorily explained by radical scavenging, suggesting that they may be the result of other, still poorly understood non-redox CNPs properties. For example, it has been proposed that CNPs dissolution occurring in particular conditions such as, e.g., acidic environment at pH ≤ 4 (Schwabe et al., 2014) or irradiation in water media (Asghar et al., 2017), may exert noxious effect, through liberation of the cytotoxic Ce^4+^ ions (Huang et al., 2010); an additional effect of the acidic environment is the inhibition of the catalase-mimetic activity of CNPs while the SOD-mimetic ability is preserved: this would lead to accumulation of H_2_O_2_, more toxic than superoxides, and a paradoxical oxidative stress (Perez et al., 2008). Such interpretations, however, must face the fact that on one side, pH > 4 does not cause CNPs alterations, and on the other, tissues and cells, though stressed or transformed, would collapse at pH ≤ 4: therefore, alternative explanations are required to understand the mechanism of the non-redox activities of CNPs.

A straightforward method to assess which CNPs actions depend on the redox activity, is probing the system with CNPs doped with Sm, a lanthanide with stable 3+ valence that substitutes Ce^3+^ ions in the lattice, pinning Ce valence at 4+ (Celardo et al., 2011a; Caputo et al., 2015). This way, the Ce^3+^/Ce^4+^ redox switch is eliminated, whereas the surface oxygen vacancies necessary to compensate the lack of +4 valence in CNPs are preserved. Upon Sm doping, we could demonstrate that the ability of CNPs to inhibit apoptosis in cells of blood origin is due to the redox switch (Celardo et al., 2011a; Caputo et al., 2015, 2017), whereas the impairment of neuronal cell differentiation is not (Gliga et al., 2017): this indicates that other features of CNPs, additional to the redox switch, may exert redox-independent biological activity.

In this study, we describe two novel unexpected findings, showing that CNPs, in addition to act as radio-protecting agents, are able to: (1) act as radio-sensitizers by restoring damage-induced intracellular checkpoints and apoptosis competence; and (2) exert this action with a mechanism independent from the Ce^3+^/Ce^4+^ redox switch.

## Materials and Methods

### CNP and SDC Synthesis and Characterization

Cerium oxide nanoparticles (CNPs) and Sm-doped Ceria (SDC) were synthesized and characterized as follows. Briefly, Pluronic F-127 (Sigma-Aldrich) surfactant was dissolved in 300 mL of deionized water at a concentration of 0.8 g mol^-1^. After 1 h, 12.4 g of cerium (III) nitrate hexahydrate (Ce (NO_3_)3 6H_2_O) or a mixture of Ce(NO3)3 × 6H_2_O and Sm(NO_3_)3 × 6H_2_O (Aldrich, Milano, Italy) in the right stoichiometric proportion were poured into the solution, followed by the addition of 13 mL of N,N,N0,N0-tetramethylethylenediamine (TEMED) (Aldrich, Milano, Italy). The solution was kept overnight under mild stirring. The precipitated gel was collected by micro filtration, rinsed repeatedly with deionized water and placed in oven at 80°C. The dehydrated material was grounded in an agate mortar and then annealed overnight at 450°C for 4 h. The CNPs or SDC obtained were characterized by powder X-Ray diffraction (XRD) and transmission electron microscopy (TEM) analysis. Phase and morphology of the materials were analyzed using an XRD diffractometer (Philips X-Pert). Nanoparticle size was determined using a TEM (FEI Titan G2 60-300 ST Cs-Image corrected) microscope. Specific surface area measurements (BET analysis) were performed treating the samples in helium flux at 300°C for 1 h using a Micromeritics Gemini V equipment.

Details and figures are available in **Supplementary Material**.

A stock dispersion of CNPs or SDC were prepared in deionized water at the concentration of 20 mg/mL. NPs were dispersed with ultrasounds (Branson Ultrasonic Corp., Danbury, CT, United States) at 20% amplitude for 5 min, and immediately diluted at the final concentration of 200 μg/mL in fresh medium. Nanoparticles were added to the cultures 1 h prior to all irradiations.

The dose was selected according to results described in Celardo et al. (2011a), where it was shown that a plateau was reached at the concentration of 200 μg/mL.

### Cell Culture

HaCat cells, a non-tumorigenic, spontaneously transformed human keratinocyte cell line, were grown at 37°C in a 5% CO2 humidified atmosphere in DMEM (4.5 g/liter glucose, 10% fetal calf serum, 100,000 units/liter penicillin, 50 mg/L streptomycin, and 200 mM glutamine). Initial seeding densities or longer growth periods are chosen depending on experimental requirements. Depending on the end point to be studied, HaCaT cells are seeded in different types of plastic flasks. Such cells were selected because they lack proper DNA damage response (DDR, mimicking in this sense a cancer system), and are a reference model for X-rays response (Boukamp et al., 1999).

### Cell Irradiation

Cells were irradiated at 80% confluence with 0.1, 1, 5, or 7 Gy of X-rays generated by CHF 320G generator (Gilardoni, Mandello del Lario, Italy) equipped with a Cu filter of 0.5 mm, operating at 250 keV, 5 mA, delivering a dose rate of 0.11 Gy/min.

### Reagents

Dihydroethidium (DHE), dichlorofluorescein (DCF), regular melting point agarose, low melting point agarose, and all the other chemicals were purchased from Sigma-Aldrich (St. Louis, MO, United States). Stock solutions: DHE (5 mM) and DHR (10 mM) were dissolved in dimethyl sulfoxide (DMSO).

### Quantification of GSH

Glutathione (GSH) levels were determined in cell lysates by high-performance liquid chromatography-mass spectrometry (HPLC-MS). Briefly, HaCat cells were lysed in aqueous solution containing 10 mM N-ethylmaleimide (Sigma-Aldrich, St. Louis, MO, United States), and kept on ice for 30 min. After centrifugation, the protein content was measured in the supernatants according to Bradford. Thereafter, 20 mM thiosalicylic acid (TSA) (Sigma-Aldrich, St. Louis, MO, United States) was added, as internal standard, and the mixture was kept on ice for another 20 min to allow the derivatization of TSA. Acetonitrile was added to precipitate proteins. After centrifugation at 10,000 *g* for 5 min, supernatants were injected and analyzed by a combined HPLC-MS system (Agilent Technologies, Palo Alto, CA, United States). The mean value of three measurements is given as GSH in nanomoles per milligram of total proteins ± SD.

### Assessment of Cell Membrane Fatty Acid Concentrations

Cell pellets were extracted twice in chloroform/methanol (2/1, v/v) in the presence of 50 μg butylated hydroxytoluene as antioxidant and 25 μg of tricosanoic acid methyl ester as internal standard. Chloroform extracts were dried under nitrogen. Fatty acids of cell total lipid extract were trans-methylated with sodium methoxide (15% w/v) in methanol and analyzed by gas chromatography-mass spectrometry (GC-MS) on a capillary column (FFAP, 60 m × 0.32 μm × 0.25 mm, Hewlett Packard, Palo Alto, CA, United States). The results were calculated after time integration of the chromatogram and final processing of areas. The identity of each fatty acid was obtained by comparing the mass spectrum of a standard mixture of fatty acids (Sigma-Aldrich, St. Louis, MO, United States). Results are given as mean of three different lipid extractions ± SD.

### Detection of ROS

Reactive oxygen species (ROS) were measured in a 96-well plate assay using the fluorescent probe dihydrodichlorofluorescein diacetate (H2DCFDA), which is de-acetylated upon cell internalization; oxidation to DCF by the cell environment (preferentially peroxides) renders the probe fluorescent. HaCat cells were incubated with 10 μM H2DCFDA in complete medium for 20 min at 37°C. DCF fluorescence was measured at 5 min after irradiation and was analyzed using a Victor plate reader set at an excitation wavelength of 485 nm and emission wavelength of 535 nm.

### Detection of Superoxides

Superoxides were assayed using 5 μM DHE (excitation 370 nm/emission 420 nm), which is sensitive to oxidation by superoxide. DHE was added directly to the cell samples after irradiation and incubated at 37°C in the dark for 20 min; then 20,000 cells for each sample were detached and analyzed by FACSCalibur flow cytometer. Data are analyzed with WinMdi 2.9 software.

### Catalase Activity

Catalase activity was measured by spectrophotometrically monitoring the rate of disappearance of 10 mM hydrogen peroxide at 240 nm (Aebi, 1984). One unit of Cat was defined as the amount of enzyme that degrades 1 μM of H_2_O_2_. Standard curves were performed using human Cat at different concentrations. The mean of three different measurements was calculated and the results are given as units of Cat per milligram proteins ± SD.

### Comet Assay

Comet assay is a single-cell gel electrophoresis method that allows detecting DNA breaks (Giovanetti et al., 2008). Alkaline comet assay permits to detect both single and double strand brakes whereas neutral comet assay allows to selectively detect double strand breaks (DSBs). One hour after irradiation (unless otherwise stated) cells were suspended in 0.5% low melting point agarose then pipetted onto a frosted glass microscope slide pre-coated with a layer of 0.2% normal melting point agarose. Slides were incubated in the alkaline lysis solution for 1 h. After lysis, slides were rinsed with electrophoresis buffer for 20 min to allow DNA unwinding. Alkaline comet assay electrophoresis buffer was prepared dissolving in deionized water 2.5 M NaCl; 100 mM EDTA; 10 mM Trizma base, and NaOH to reach pH 10, while neutral comet assay electrophoresis buffer was made dissolving Tris-Base (2 M), Acetic acid (1 M), and EDTA (50 mM) to reach pH 8. Electrophoresis was conducted for 30 min at 20 V with 300 mA in a unit Sub cell GT System/15 cm × 25 cm system equipped with Power Pack 300 (Bio-Rad Laboratories Inc., Hercules, CA, United States). In alkaline comet assay, slides were then gently washed in neutralization buffer solution for 5 min. This step was not necessary in the neutral Comet procedure. Then, slides were dehydrated with ethanol series, and dried at room temperature. One hundred cells on each slide were scored using a fluorescence microscope; the extent of genetic damage was evaluated by visual scoring provided in arbitrary units after validation by comparison with computer image analysis (García et al., 2004).

### Cell Cycle Analysis

Cell suspensions were washed with PBS and fixed overnight in ethanol 70% at -20°C, treated with RNAse at 200 μg/mL, stained with PI at the final concentration of 50 μg/mL and finally 20,000 cells for each sample were analyzed by FACSCalibur flow cytometer. Data were analyzed with WinMdi 2.9 software. Cells population in G1 and in G2 phases were estimated by gating the area of the relative peaks, and G2/G1 ratio was calculated.

### Evaluation of Apoptosis

Apoptosis was evaluated quantifying the fraction of apoptotic nuclei by fluorescence microscopy after DNA staining with the cell-permeable specific dye Hoechst 33342, directly added to the cell culture. To evaluate the eventual presence of necrotic cells, cells were also stained with PI at a final concentration of 5 μg/mL. The fraction of apoptotic nuclei among the total cell population was calculated by counting at the fluorescence microscope at least 300 cells in at least three randomly selected microscopic fields (Ghibelli et al., 1995).

### Clonogenic Cell Survival Assays

Immediately after exposure to X-rays cells were trypsinized. After cell quantification with a Burker counting chamber, duplicate 60-mm tissue culture dishes were seeded with aliquots of 200 cells/mL each. Colonies were allowed to form for 14 days, after which they were fixed with methanol and stained with crystal violet.

### Micronuclei Assay

The micronuclei test is a broad-spectrum mutagenesis test (Furness et al., 2010). Micronuclei are small nuclear bodies arising from improper chromosome separation at mitosis as a consequence of chromosomal lost or mis-repaired DNA damage. Evaluation of the number of micronuclei among cells undergoing the mitotic telophase is a measure of early mutagenesis after genotoxic treatments. After irradiation, cytochalasin B (3 μg/mL; Sigma-Aldrich) was added to the cells to prevent cell division without inhibiting mitosis. After 24 h, the resulting bi-nucleated cells label those that underwent mitosis. Then the medium was removed, the cells were rinsed with PBS, treated with hypotonic solution (KCl 0.075M) for 3 min and then fixed by Carnoy fixative (methanol/acetic acid, 20:1) for 8 min and then stained with Hoechst 33342. A total of 500 bi-nucleated cells were scored under a fluorescent microscope; the values given in the graphs represent the number of micronuclei per 500 bi-nucleated cells.

### Statistical Analysis

Each experiment was repeated ≥3 times. Data are presented as means ± SD. Statistical evaluation was conducted by a one-way ANOVA, followed by Tukey’s Multiple Comparison Test (Homogeneous Variances) using the software Origin 8.0. Statistical significance was set at *p* < 0.05.

## Results

### CNPs Decrease X-Ray-Induced Oxidation and DNA Damage

**FIGURE 1:**
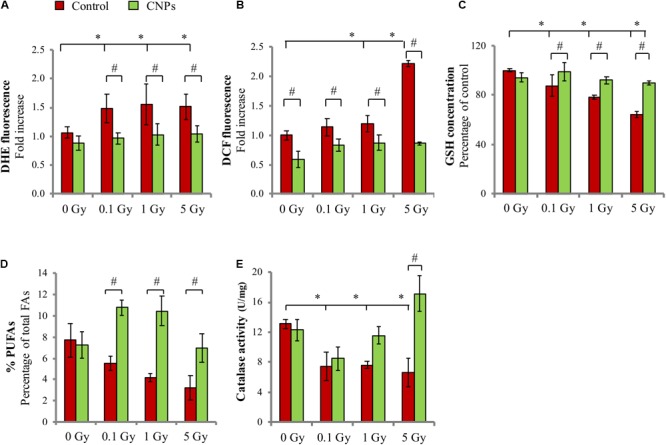
Cerium oxide nanoparticles (CNPs) protect HaCat cells from X-ray-induced redox imbalance. **(A)** DHE and **(B)** DCF fluorescent signal fold increase with respect to the control detected 1 h or 5 min after irradiation at 0.1, 1, or 5 Gy, respectively. Intracellular glutathione, GSH **(C)** and membrane poly-unsaturated fatty acids (PUFAs) levels **(D)** measured 1 h after irradiation at 0.1, 1, or 5 Gy in cells. **(E)** Catalase activity measured 1 h after irradiation with 0.1, 1, or 5 Gy in cells. Values are the mean of ≥3 experiments ±SD; ^∗,#^*p* < 0.05 (ANOVA). Significance of all the mean groups with respect to the control group (^∗^) and (#) comparison between single mean groups ±CNPs irradiated with the same X-rays dose are shown.

HaCat cells irradiation with 0.1, 1, and 5 Gy of X-rays increases intracellular superoxides (DHE signal, **Figure 1A**) and peroxides (DCF signal, **Figure 1B**), measured as fluorescence emitted by the relative probes when oxidized. The increase is inhibited by CNPs, which maintain the values of both types of ROS at basal, i.e., pre-irradiation, levels. Among the consequences of ROS increase, **Figure 1C** shows that X-ray irradiation dose-dependently and significantly decreases the levels of intracellular GSH, the most abundant endogenous molecular antioxidant responsible for preserving a reduced cytosolic redox state and protein conformation (Filomeni et al., 2002), and almost depletes cell membranes of poly-unsaturated fatty acids (PUFAs), the main reservoir of oxidizable double-bonds protecting membrane integrity from oxidative stress (**Figure 1D**). CNPs restore GSH and PUFAs concentration to levels similar to untreated cells. Importantly, CNPs also restore the intracellular catalase activity that was inhibited by irradiation (**Figure 1E**), without affecting protein level (controlled by Western blotting, data not shown), showing that CNPs exert a potent antioxidant action on the irradiated cells.

**FIGURE 2:**
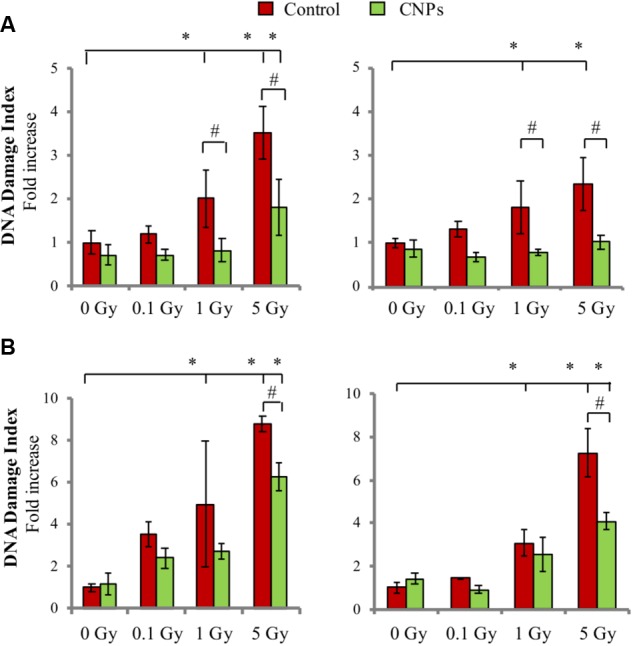
Cerium oxide nanoparticles protect from DNA damage and accelerate DNA repair. SSB and DSB quantification evaluated by alkaline **(A)** or neutral **(B)** comet assay 1 h (Left) or 24h (Right) after X-rays irradiation ±CNPs. Values are the mean of ≥3 independent experiments ±SD, and are normalized for values of untreated cells; ^∗,#^*p* < 0.05 (ANOVA). Significance of all the mean groups with respect to the control group (^∗^) and (#) comparison between single mean groups ±CNPs treated with the same X-rays dose are shown.

Another major consequence of X-ray irradiation is damage to DNA, which is especially sensitive to breaks to the sugar-phosphate chain induced directly by the ionizing radiation, forming DSB, and indirectly by radiation-induced ROS, forming single strand breaks (SSBs) (Barcellos-Hoff et al., 2005; Borrego-Soto et al., 2015). Here, we report that X-rays dose-dependently produced early SSB (**Figure 2A**) and DSB (**Figure 2B**) in HaCat cell DNA, as assessed by alkaline or neutral comet assay, respectively, at 1 h post-irradiation. CNPs strongly protected from SSB formation, maintaining basal levels at all doses. CNPs exerted only a partial protection against DSB, possibly dealing with the fraction of DSB resulting from vicinal SSB formed in opposite DNA strands, which are conceivably of an oxidative origin. CNPs accelerate DNA repair (**Figure 2**), confirming what previously reported (Caputo et al., 2015), completely sealing SSB at all doses after 24 h. Notably, CNPs act as antioxidant agents also in non-irradiated cells, slightly reducing oxidative parameters and basal DNA breaks.

### CNPs Sensitize HaCat to Radiation-Induced Cell Death

**FIGURE 3:**
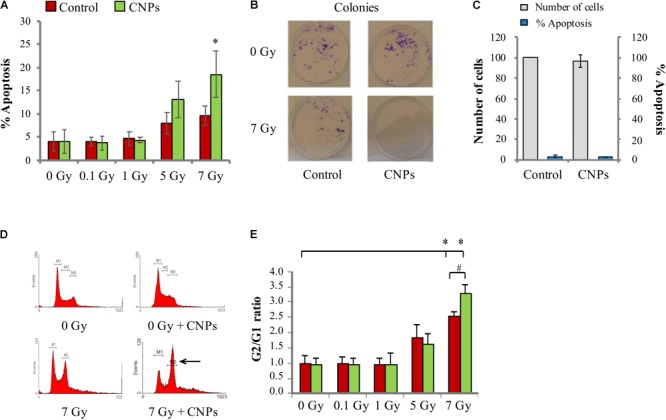
Cerium oxide nanoparticles increase cell response to irradiation. **(A)** Percentage of X-ray-induced apoptosis on HaCat cells 48 h after irradiation at 0.1, 1, 5, or 7 Gy. **(B)** Number of colonies formed by HaCaT cells irradiated at 7 Gy ± CNPs. **(C)** Proliferation (gray) and percentage of apoptosis (blue) of cells treated or not with CNPs for 24 h. **(D)** Cell cycle profiles of HaCaT cells 24 h after irradiation at 0–7 Gy ± CNPs. **(E)** Dose dependence of G2/G1 ratio measured at 24 h after irradiation at 0.1, 1, 5, or 7 Gy. Values are the mean of ≥3 independent experiments ±SD; ^∗,#^*p* < 0.05 (ANOVA). Significance of all the mean groups with respect to the control group (^∗^) and (#) comparison between single mean groups ±CNPs irradiated with the same X-rays dose are shown.

X-rays promoted apoptosis on HaCat cells, starting at 5 Gy (**Figure 3A**); an additional intensity at 7 Gy allowed showing a dose-dependent response. Thus, while DNA damage is increasingly induced by all doses, it is able to promote apoptosis only at the higher intensities, suggesting a high threshold level of damage perception for HaCat. Surprisingly, CNPs not only failed to reduce radiation-induced apoptosis, as expected due to their ability to reduce the damage, but even incremented it at 5 and 7 Gy.

To verify the role of CNPs on survival of HaCat to radiation damage, we measured the ability of irradiated HaCat cells to form colonies in the presence and absence of CNPs (clonogenicity assay). As shown in **Figure 3B**, X-rays strongly reduced colony formation, as expected. The reduction is exacerbated by CNPs: this supports the data on apoptosis, confirming that CNPs reduce cell survival to damage, even in a long time-frame, in spite of their damage-protective effects.

The possibility that the increment of apoptosis may be due to a generic toxic effect of CNPs on HaCat was excluded, because in non-irradiated cells, CNPs poorly affected clonogenicity or apoptosis, or the rate of HaCat cell proliferation (**Figure 3C**). These data are rather compatible with a scenario where CNPs selectively affect features specifically induced by irradiation, possibly restoring apoptosis competence by non-toxic mechanisms, being in fact radio-sensitizing agents.

With this in mind, we investigated whether CNPs could restore a DNA damage-induced response responsible for apoptosis competence. Cells respond to DNA damage by setting up responses aimed at repairing the damage (DDR) or induce apoptosis (Caputo et al., 2012). This implies blocking cell cycle in the G2 phase (i.e., before mitosis), to give time to complete repair before cell division, avoiding irreversible genetic damage. If repair cannot be accomplished according to the intrinsic integrity checkpoints, cells push themselves to apoptosis. This is a major cancer preventive mechanism; accordingly, in cancer cells the perception of DNA damage, the onset of DDR, and the propensity for the apoptosis outcome are impaired, causing mutation permanence and tumor progression (Hanahan and Weinberg, 2000). So, we investigated whether CNPs radio-sensitization may imply an improvement of the DDR.

We measured the cycle of irradiated HaCat by standard flow cytometric analysis of DNA content. **Figure 3D** shows the profiles of cell cycles at 0 and 7 Gy, visually showing the abundance of cells in each cell cycle phase. **Figure 3E** shows the calculated abundance of cells in the G2 phase (as G2/G1 ratio), demonstrating that irradiation at 0.1 and 1 Gy does not affect cell cycle, whereas 5 and 7 Gy dose-dependently increases cell cycle pausing in G2. Since in our system DSB are produced also by the lowest doses (**Figure 2**), this means that HaCat have a very high DNA damage threshold level for DDR activation, behaving in this regard like most cancer cells do. In comparison, in normal fibroblasts the cell cycle arrest is complete at radiation intensities as low as 0.1 Gy (Yang et al., 2005). CNPs do not affect the pause at 5 Gy, but at 7 Gy they significantly increase the block in G2. The extent of cell cycle blockade depends on the severity of the damage on one side, and on the strength of the cellular response, which is a function of the peculiar asset of specific cells, on the other. Since CNPs reduce radiation damage, this indicates that CNPs act to potentiate the DDR, leading to the apparent paradox of increased response to a reduced damage.

Summarizing, while the radio-protective effect of CNPs deals with providing an extra antioxidant defense to irradiated cells, thus being effective at any intensity of irradiation strong enough to damage cells, the radio-sensitizing effect consists in the magnification of the cell response to DNA damage favoring the apoptotic outcome, indicating that different functions of CNPs may be responsible for these different effects.

### CNPs Radio-Sensitizing Ability Occurs Through Oxidation-Independent Mechanisms

The finding that CNPs increase radio-induced apoptosis argues against a SOD-mimetic effect, because SOD is frankly anti-apoptotic (Zhang et al., 2008). Apoptosis impairment is not limited to SOD, but a general effect of antioxidant enzymes [e.g., catalase (Scheit and Bauer, 2014)]: therefore, this raises concerns about the fact that the radio-sensitizing effect of CNPs is actually a redox issue.

To investigate this point, we used a nanotechnology tool that allows discriminating redox vs. non-redox activity of CNPs. We have previously shown that Sm-doped CNPs (SDC), with size and shape similar to the pristine CNPs, are unable to scavenge ROS or to protect cells from oxidative insult due to the loss of the Ce^3+^/Ce^4+^ redox switch (Celardo et al., 2011a; Caputo et al., 2015).

**FIGURE 4:**
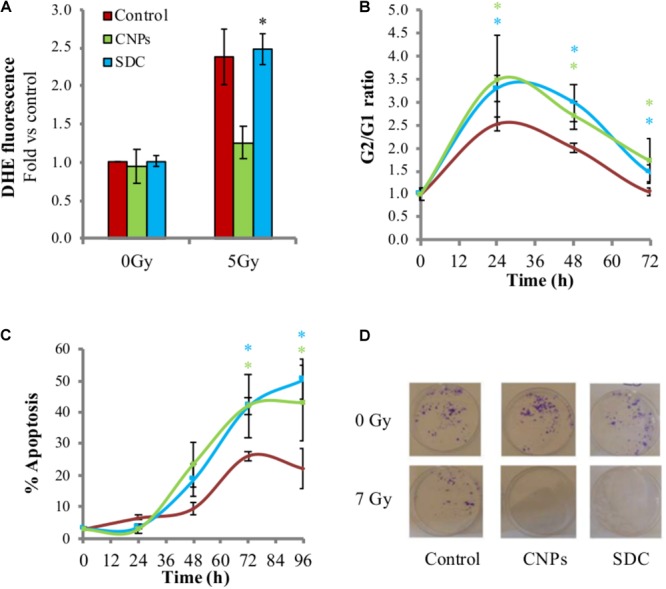
Cerium oxide nanoparticles radio-sensitization is independent from their redox activity. **(A)** Superoxide levels measured by DHE fluorescent signal detected by flow cytometry 1 h after irradiation ±CNPs or SDC at 200 μg/ml. **(B)** Kinetic of cell cycle arrest in cells irradiated at 7Gy ± CNPs or SDC. **(C)** Time course of X-rays-induced apoptosis on HaCat cells 24–96 h after irradiation at 7 Gy ± CNPs or SCD. Values are the mean of ≥3 independent experiments ±SD; ^∗^*p* < 0.05 (ANOVA). Comparison between single mean groups ±CNPs or SDC irradiated with the same X-rays dose are shown. **(D)** Colonies (violet spots) formed by HaCaT cells irradiated at 7 Gy ± CNPs or SDC.

Here the effects of SDC on irradiated HaCat were tested, and it was observed that they fail to prevent X-ray-induced superoxide formation (**Figure 4A**), confirming the lack of antioxidant effect. SDC were then compared with CNPs for the radio-sensitizing effects. Interestingly, SDC maintained the ability of CNPs to increase cell cycle arrest (**Figure 4B**) and apoptosis (**Figure 4C**), and to reduce colony formation (**Figure 4D**), to the same extent as the un-doped nanoparticles do. This indicates that some features of CNPs, different from the well-recognized antioxidant effect due to the Ce^3+^/Ce^4+^ redox switch, exert a potent biological effect responsible for radio-sensitization, enhancing DNA damage perception and apoptotic outcome.

### CNPs and SDC Nearly Abrogate X-Ray-Induced Mutagenesis

**FIGURE 5:**
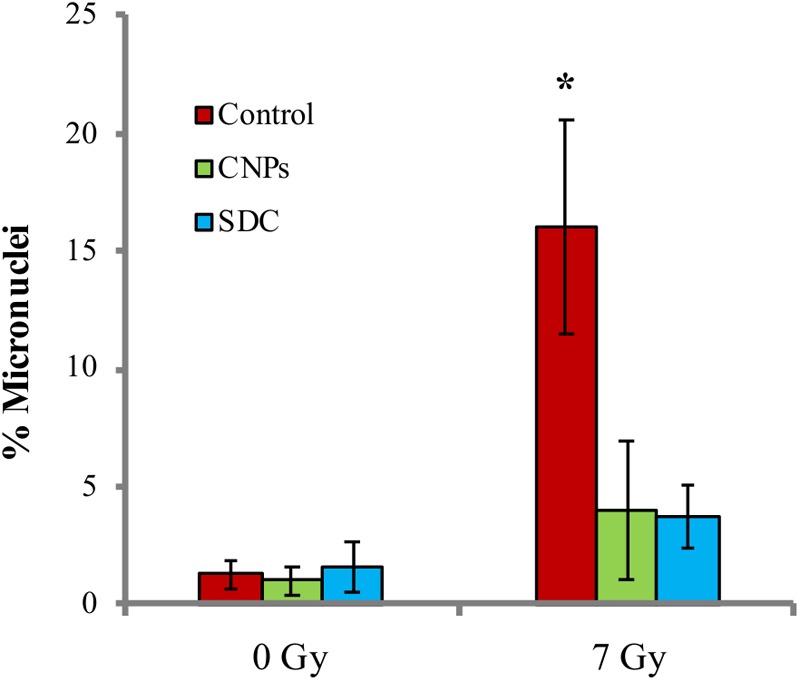
Cerium oxide nanoparticles prevent radiation-induced mutagenesis. Percentage of micronuclei among bi-nucleated cells 24 h after irradiation at 7Gy ± CNPs. Values are the mean of ≥3 independent experiments ±SD; ^∗^*p* < 0.05 (ANOVA). Significance of all the mean groups with respect to the control group is shown.

Damaged cells escaping the cell cycle block in G2, undergo mitosis before repair, and this may render permanent an altered genetic asset caused by the damage, thereby promoting mutagenesis. We analyzed mutagenesis by performing the micronuclei assay, a single cell analysis that allows detecting a broad range of genetic alterations, by assessing the frequency of cells containing extra, small nuclear bodies. A peculiar advantage of this technique is the possibility of separately evaluating micronuclei among cells that underwent mitosis, which are recognizable for being bi-nucleated, as cytokinesis is blocked by cytochalasin (Furness et al., 2010): in case of irradiation, bi-nucleated cells identify those that escaped the cell cycle block in G2. As shown in **Figure 5**, X-rays increase the fraction of micronucleated cells, indicating a pro-mutagenic effect of irradiation, as expected. CNPs and SDC sharply contrast this phenomenon, nearly abolishing mutagenesis.

Decreased mutagenesis may be ascribed to decreased DNA damage on one side, and increased stringency of cellular integrity checkpoints that eliminate highly damaged cells, on the other. CNPs reduced early radiation damage, whereas SDC did not: thus, the fact that SDC and CNPs equally reduce radiation-induced mutagenesis, implies that decreased damage is poorly relevant, pointing instead to increased stringency of the checkpoints as the major determinant for the CNPs-mediated maintenance of genetic integrity.

## Discussion

The main messages from this study are that CNPs are able to act as radiotherapeutic-sensitizing agents by reinforcing the stringency of the cellular DNA integrity checkpoints, which is a potent cancer preventive process; and that this effect is driven by a novel, non-redox activity of CNPs.

Radio-sensitization is a major goal in radiotherapy because cancer cells, which should be ideally killed by the highly toxic ionizing radiations used in clinical practice, are instead generally resistant, often evading radiation-induced apoptosis. Therefore, agents or processes that help inducing or incrementing death of irradiated cancer cells may improve the therapeutic outcome, and are highly searched for in prospective clinical application.

“Biological” radio-sensitization may be achieved by interfering with cell survival or apoptotic pathways. For example, DNA repair inhibition hampers survival of DNA-damaged cells that cannot seal the induced lesions, increasing radiation-induced apoptosis; such strategies are receiving much attention nowadays (Ďurišová et al., 2018), even though they do not involve selectivity against cancer cells. A more stringent approach implies specifically hitting intrinsic features of the target cancer cells (e.g., sloppy DNA integrity checkpoints or resistance to apoptosis) during irradiation. Radio-sensitizing agents acting this way have the advantage of increasing cell death selectively in cells with poor apoptosis competence, i.e., cancer cells, sparing the normal cells present in the irradiated area.

Treatments generically allowing achieving a higher level of cancer cell killing with respect to radiation alone (independently of the selectivity), also are considered to be radio-sensitizers. Bioactive nanoparticles, including CNPs, possess several of such radio-sensitizing properties. Nanoparticles made of high atomic number materials, including CNPs, when irradiated with specific energy beams (*n.b.*, different from that used in our study), emit toxic ROS or heat (“dose-enhancement effect”), thus resulting in greater toxicity than irradiation alone, on any cells present in the irradiated area (Misawa and Takahashi, 2011; Caputo et al., 2014). It was reported that CNPs exert on leukemic HL60 cells toxic effects on their own [due to still unexplained mechanism, see (Corsi et al., 2018)], which sum up with that induced by irradiation (Montazeri et al., 2018): this is actually an additional toxicity effect rather than *bona fide* radio-sensitization. In a further study showing *in vitro* radio-sensitization of pancreatic cancer cells by CNPs, it was proposed that the acidic environment of the irradiated cells may impair the catalase-mimetic activity of CNPs, causing H_2_O_2_ accumulation and toxicity (Wason et al., 2013); however, this explanation do not consider that to inhibit the catalase mimetic CNPs activity it is necessary to reach pH ≤ 4, values not compatible with what is found in tissues, even cancerous or irradiated (Corsi et al., 2018). In any case, in our system CNPs not only do not impair catalase activity, but rather, they even restore it when it is destroyed by X-rays, abating ROS formation.

In this study, we describe a CNPs radio-sensitizing activity that does not involve any of the above described mechanisms. Rather, we provide evidence that CNPs are radio-sensitizers in the most stringent sense. On one side, their action is only perceived by cells that are damaged in their DNA, because CNPs were ineffective on non-irradiated HaCat. On the other, they restore apoptosis competence on HaCat cells, which possess a weak DNA damage integrity checkpoint, suggesting that they may have no effect on non-transformed cells equipped with a robust DDR of their own.

The scenario of our *in vitro* system is reminiscent of cancer cells (with their impaired checkpoints) treated with DNA damaging radio-therapeutic sources of ionizing radiations. The cellular targets of CNPs radio-sensitizing action therefore should be those molecular determinants deployed to the restoration of a DNA integrity checkpoint, responsible for reinforcing both the perception of DNA damage, and the proneness to the apoptotic outcome in case of mal-repair. The DDR is a very well-studied signal transduction process, with complex and branched interactions (Caputo et al., 2012), which is often malfunctioning in cancer cells, allowing cell duplication in spite of DNA damage. This causes accumulation of mutations, constituting the molecular bases of cancer progression. A sloppy DNA integrity control may be due to loss or gain of function of specific activators or repressors of DDR, respectively. So, we may speculate that CNPs may either restore or supply a DDR activator function lost in cancer cells, or inhibit an overexpressed signaling pathway aimed at bypassing DDR. The latter is an event that often occurs in the inflammation conditions that predispose to cancer (Ara and Teicher, 1996; Shureiqi and Lippman, 2001), and implies concerted actions preserving cell viability even in the presence of damage (Kidane et al., 2014). We are actively working in this direction to identify possible CNPs target(s) allowing bypassing such deleterious cell survival pathways.

Cerium oxide nanoparticles are considered very promising agents in antioxidant therapy, due to their peculiar auto-regenerative antioxidant mechanism (Celardo et al., 2011c). The original aim of this study was to investigate the antioxidant mechanism of CNPs radio-protective effects at the cell and tissue level, considering that cancer microenvironment is deeply affected by redox imbalance (Helfinger and Schröder, 2018; Hegedûsa et al., 2018). The technique of Sm doping was indeed adopted to rule out any other possible effects of CNPs, being in fact originally used as negative control (Celardo et al., 2011b). Surprisingly, however, this tool has allowed pointing out that unexpected non-redox bioactivity of CNPs (Gliga et al., 2017 and this study).

Non-redox activities of CNPs, with different mechanisms, have been described. They include CNPs dissolution, a phenomenon occurring in highly acidic (pH ≤ 4) environments (Schwabe et al., 2014), leading to release of toxic Ce^4+^ ions (Huang et al., 2010). It is reported that ionizing irradiation in aqueous environment can strongly promote ceria dissolution via acidification, with release of Ce^4+^ ions (Asghar et al., 2017), hence inducing toxicity. Though potentially logically explaining the selective toxicity against irradiated cells we describe here, some considerations argue against this mechanism as an explanation for our results. Indeed, acidic events leading to CNPs dissolution (pH ≤ 4) would have been easily perceived, being visually detectable through the pH-sensitive dye included in the standard culture media formulations. Moreover, such levels of acidification are incompatible with cell functions or survival (Corsi et al., 2018). Therefore, it remains to be understood which is the non-redox feature of CNPs responsible for the radio-sensitizing effect reported here. We cannot exclude that irradiation may re-localize internalized CNPs into the lysosome acidic cell compartment, which have a pH compatible with CNP dissolution. This may have the consequence of selectively producing a toxic mediator such as Ce^4+^ ions, previously reported to increase the toxic effect of radiations (Floersheim, 1995), at the very moment when an apoptotic outcome would be welcome as a response to DNA damage. If such a mechanism is compatible with a reduced cell survival to stress, it hardly explains, however, the improved perception of the DNA damage leading to increased cell cycle blockade and apoptosis: therefore, other mechanisms should be investigated to fully understand the role played by CNPs in increasing the stringency of the cell integrity checkpoints.

## Conclusion

This study highlights novel perspectives to the actions of CNPs as anticancer and in general medicinal devices, describing new functions in the control of cell signaling. This contributes to the emerging evidence that CNPs may exert a bio-modulatory control of cancer microenvironment, re-establishing apoptosis competence and possibly participating to the anakoinosis communicative reprogramming process that is implicated in the pharmacological restoration of normal homeostasis in cancer tissues (Hart et al., 2015). Overall, our findings pose new questions that we hope will be answered in a reasonable time frame.

## Author Contributions

FaC and LG wrote the paper. FaC made most cell experiments. FrC performed the experiments and helped with manuscript preparation. VM did the redox measurements and interpreted the results. SB performed PUFA analyses and interpreted the results. AG did all the X-rays exposure and supervised the biological experiments. ET and SL supervised all nanoparticle issues. LG coordinated the project. All authors participated in the development of the scientific work, contributed to discussion on experiments and presentation, and approved the manuscript.

## Conflict of Interest Statement

The authors declare that the research was conducted in the absence of any commercial or financial relationships that could be construed as a potential conflict of interest.
