# Supplementary material for: Cerium Oxide Nanoparticles Re-establish Cell Integrity Checkpoints and Apoptosis Competence in Irradiated HaCat Cells via Novel Redox-Independent Activity
Source: Front Pharmacol. 2018 Oct 16;9:1183. doi: 10.3389/fphar.2018.01183 (PMC6232693; doi:10.3389/fphar.2018.01183)
Supplement: Supplementary file 1 [file Table_1.docx]

Supplementary Material

Cerium oxide nanoparticles re-establish cell integrity checkpoints and apoptosis competence in irradiated HaCat cells *via* novel redox-independent activity

Fanny Caputo*^1,2^; Anna Giovanetti^3^; Francesca Corsi^1^; Vittoria Maresca^4^; Stefania Briganti^4^; Silvia Licoccia^1^; Enrico Traversa*^5^ and Lina Ghibelli*^1^

^1^Department of Chemical Science and Technologies, University of Rome Tor Vergata, Rome, Italy

^2^Department of Biology, University of Rome Tor Vergata, Rome, Italy

^3^ENEA SSPT-TECS-BIORISC, Rome, Italy

^4^San Gallicano Dermatological Institute IRCCS, Rome, Italy

^5^ School of Materials and Energy, University of Electronic Science and Technology of China, 2006 Xiyuan Road, Chengdu 611731, Sichuan, P. R. China

*** Correspondence:**Enrico Traversa
traversa@uestc.edu.cn

Lina Ghibelli

ghibelli@uniroma2.it

Fanny Caputo
fanny.caputo@gmail.com


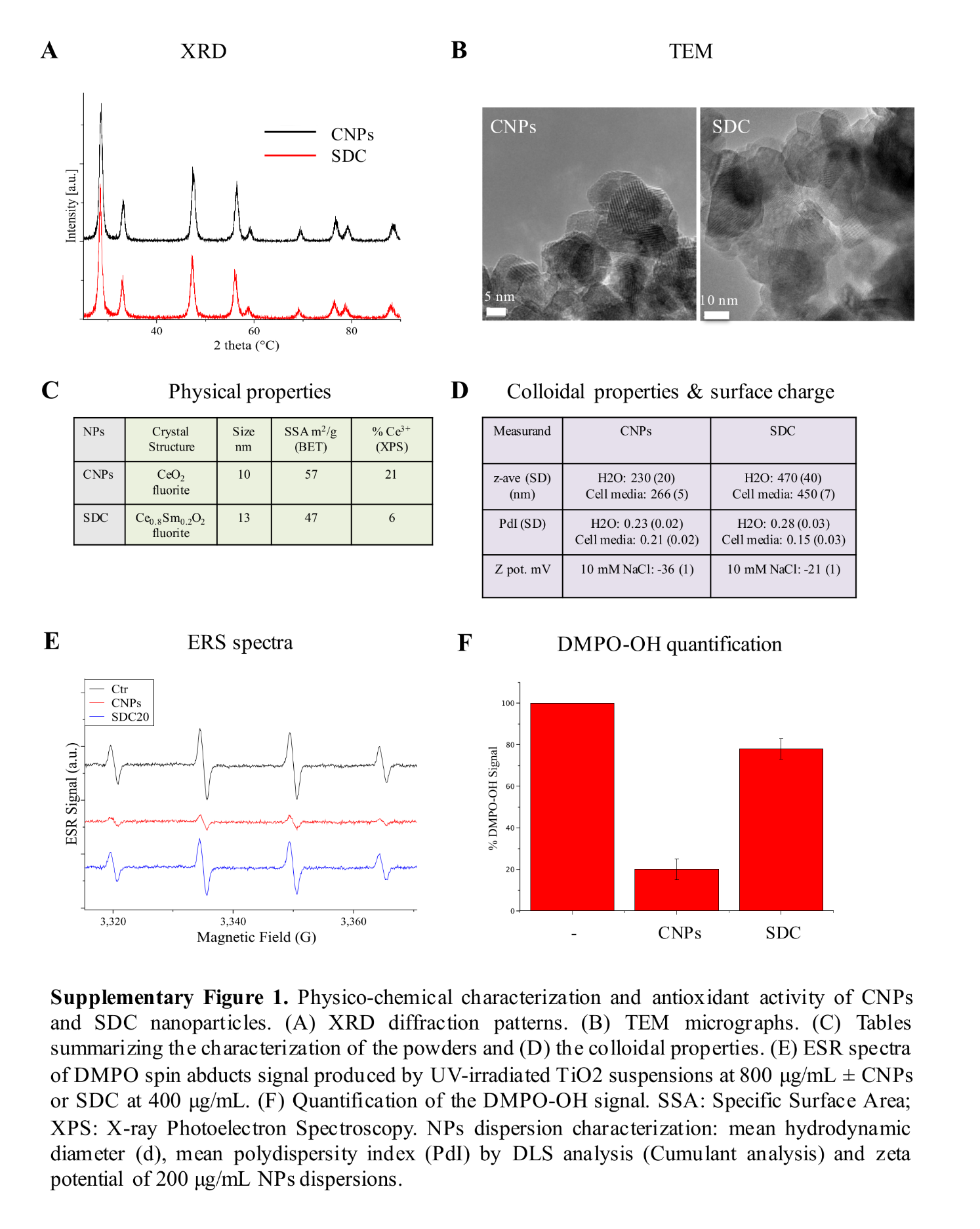


**Supplementary Figure 1**. Physico-chemical characterization and antioxidant activity of CNPs and SDC nanoparticles. (A) XRD diffraction patterns. (B) TEM micrographs. (C) Tables summarizing the characterization of the powders and (D) the colloidal properties. (E) ESR spectra of DMPO spin abducts signal produced by UV-irradiated TiO2 suspensions at 800 μg/mL ± CNPs or SDC at 400 μg/mL. (F) Quantification of the DMPO-OH signal. SSA: Specific Surface Area; XPS: X-ray Photoelectron Spectroscopy. NPs dispersion characterization: mean hydrodynamic diameter (z-ave), mean polydispersity index (PdI) by DLS analysis (Cumulant analysis) and zeta potential of 200 μg/mL NPs dispersions.

**Characterization of nanoparticles**

**Powder characterization**

The cerium oxide nanoparticles (CNPs) or Sm-doped ceria (SDC) powders obtained were characterized by powder X-Ray diffraction (XRD) and transmission electron microscopy (TEM) analysis. Phase and morphology of the materials were analyzed using an XRD diffractometer (Philips X-Pert). Nanoparticle size was determined using a TEM microscope (FEI Titan G2 60-300 ST Cs-Image corrected). Specific surface area measurements (BET analysis) were performed treating the samples in helium flux at 300°C for 1 h using a Micromeritics Gemini V equipment.

**Nanoparticle dispersions & characterization**

Stock dispersion of CNPs or SDC were prepared in deionized water at the concentration of 20 mg/mL. NPs were dispersed with ultrasounds (Branson Ultrasonic Corp., Danbury, CT, USA) at 20% amplitude for 5 minutes, and immediately diluted at the final concentration of 200 µg/mL in fresh medium. Nanoparticles were added to the cultures 1 hour prior to all irradiations. Particle size distribution was measured by dynamic light scattering (DLS) at the concentration of 200 μg/mL immediately after sonication and dilution in milliQ water or in DMEM medium + 10% fetal calf serum (FCS, to mimic cell culture environment), with a Malvern Zetasizer (Nano-ZS, Malvern Instruments, Worcestershire, UK). DLS experiments consisted of 15 runs per measurement and all experiments were carried out in triplicate. The mean of the hydrodynamic diameter and the dispersity index (P.I.) by cumulative analysis is shown ± standard deviation (SD). Zeta potential was measured in 10 mM NaCl. The experiments consisted of 100 runs per measurement and all experiments were carried out in triplicate. The mean of each triplicate measurement ± standard deviation (SD) is shown.

**Antioxidant activity of CNPs and SDC *in vitro***

CNP and SDC redox activities were tested by electron paramagnetic resonance spectroscopy (ESR) measuring the ability to scavenge hydroxyl radical. The ESR measurements were carried out at room temperature using a Bruker X-band ESR spectrometer, Bruker Spectrospin Model EleXsys 500, which was equipped with a super-high-Q cavity, Bruker Model ER 4122SHQE. HO● radicals were generated by exposing aqueous suspensions of nanoparticular TiO2 (anatase) NPs57-59 to UV-A light (***λ*ex** = 365 nm), using a UV spot light source, Lightingcure™, model LC-8 (Hamamatsu Photonics, France). During exposure to light, the 2-mL volumes of suspensions were equilibrated with oxygen at the atmospheric pressure and stirred vigorously to prevent agglomeration of nanoparticles. To avoid overheating by light, the suspensions were maintained at a stabilized temperature of 25.0 ± 0.1°C, using a Thermo Fisher Scientific Haake K10 bath vessel with a temperature control module. 5,5-dimethyl-1-pyrroline N-oxide (DMPO) was used as a spin trap of HO● radicals, thus leading to formation of ESR detectable DMPO/OH spin adducts. Prior to ESR

measurements, an aqueous dispersion of 800 ug/mL TiO2 ± 400 ug/mL CNPs or SDC was sonicated as previously described and then mixed with the stock solution of the spin trap to achieve the final DMPO concentration of 50 mM. Thus, prepared solutions were then exposed to UV-A light for determined intervals of time, while being continuously stirred using a magnetic bar. After each illumination step, the suspensions volumes of *~*15 μL were drawn into thin-walled borosilicate glass capillaries (0.7 mm ID, 0.87 mm ID, Model CV7087-100, VitroCom Inc., Mountain Lakes, NJ, USA), sealed on both ends with a tube sealant, ChaSeal (Chase Scientific Glass Inc., Rockwood, TN, USA). Control ESR measurements were always performed for all the nanoparticle suspensions prior to exposing them to light. The typical instrumental settings during these measurements were: microwave frequency 9.399 GHz, microwave power 0.64 mW, sweep width 120 G, modulation frequency 100 kHz, modulation amplitude 0.5 G, receiver gain 60 dB, time constant 41 ms, conversion time 82 ms, and total scan time ~168 s.
